# Mechanistic insights into ^125^I seed implantation therapy for Cholangiocarcinoma: focus on ROS-Mediated apoptosis and the role of GPX2

**DOI:** 10.1007/s00432-024-05840-0

**Published:** 2024-06-25

**Authors:** Jun Luo, Zheng Yao, Weiren Liang, Danjun Song, Hui Zeng, Yi Jiang, Zhehan Bao, Jiaping Zheng, Yinan Ding

**Affiliations:** Department of Interventional Radiology, Zhejiang Cancer Hospital, Hangzhou Institute of Medicine (HIM), Chinese Academy of Sciences, Zhejiang Key Laboratory of Imaging and Interventional Medicine, Hangzhou, Zhejiang 310022 China

**Keywords:** ^125^I seeds, Cholangiocarcinoma, ROS-mediated apoptosis, GPX2

## Abstract

**Objectives:**

Cholangiocarcinoma (CCA) is a rare tumor with a poor prognosis and poses significant therapeutic challenges. Herein, we investigated the mechanism of efficacy of ^125^I seed implantation therapy in CCA, focusing on the induction of reactive oxygen species (ROS)-mediated apoptosis and the involvement of glutathione peroxidase 2 (GPX2).

**Materials and methods:**

Human cholangiocarcinoma cell lines QBC939 and RBE were purchased for in vitro studies. In vivo studies were performed using a rabbit VX2 CCA model. Apoptosis and proliferation were detected by TUNEL staining and clone formation, respectively. ROS generation was detected by dihydroethidium staining. Histological evaluation was performed by hematoxylin and eosin staining. Protein expression was determined by Western blotting and immunohistochemistry.

**Results:**

Our results demonstrate that ^125^I seeds effectively inhibited tumor growth in the rabbit VX2 tumor model and promoted the apoptosis of CCA cells in vitro in a dose-dependent manner. Molecular analyses indicate a marked increase in reactive oxygen species (ROS) levels following treatment with ^125^I seeds, suggesting the involvement of ROS-mediated apoptosis in the therapeutic mechanism. Furthermore, the downregulation of glutathione peroxidase 2 (GPX2) was observed, indicating its potential role in modulating ROS-mediated apoptosis in CCA.

**Conclusion:**

^125^I seed implantation therapy exerts therapeutic effects on CCA by inducing ROS-mediated apoptosis. The downregulation of GPX2 may contribute to enhanced ROS accumulation and apoptotic cell death. These findings provide mechanistic insights into the therapeutic potential of ^125^I seed implantation for CCA and highlight ROS-mediated apoptosis and GPX2 regulation as promising targets for further investigation and therapeutic intervention in this malignancy.

## Introduction

Cholangiocarcinoma (CCA), originating from the bile duct epithelium, represents a formidable therapeutic challenge due to its aggressive nature and limited treatment options. Despite being a rare disease, global morbidity and mortality have increased over the past two decades (0.3-6 cases per 100,000 inhabitants per year in Western countries and more than 6 cases per year in some parts of East Asia) (Banales et al. 2020; Bertuccio et al. 2019). Surgical resection, the primary curative approach for localized disease, is often not feasible in advanced or inoperable cases, leaving patients with few effective treatment options and poor prognoses (Clements et al. 2020). As such, there is an urgent need for alternative therapeutic strategies that can effectively target CCA while minimizing treatment-related morbidity.

In recent years, iodine-125 (^125^I) seed implantation therapy has emerged as a promising treatment modality for various solid tumors, including CCA (Pang et al. 2019; Cheng et al. 2017). This brachytherapy technique involves the percutaneous implantation of radioactive ^125^I seed directly into the tumor tissue, allowing for precise and localized radiation delivery. Compared to conventional external beam radiation therapy, ^125^I seed implantation offers several advantages, including enhanced tumor targeting, reduced radiation exposure to surrounding healthy tissues, and improved patient tolerance (Zhang et al. 2022; Zhuang et al. 2009).

Moreover, accumulating evidence suggests that the anti-tumor mechanism of ^125^I seed implantation is to promote apoptosis and inhibit the proliferation of cancer cells (Zhou et al. 2022; Li et al. 2019a, b). Recent studies indicate that ^125^I seed induces ROS-mediated apoptosis (Wang et al. 2020; Liu et al. 2018). ROS, including free radicals and non-radical oxygen derivatives, play pivotal roles in cellular signaling and homeostasis. Excessive ROS generation can overwhelm cellular antioxidant defense mechanisms, leading to oxidative stress-induced apoptosis (Moloney and Cotter 2018). Glutathione peroxidase 2 (GPX2), a member of the GPX family, is a key antioxidant enzyme that catalyzes the reduction of hydrogen peroxide and organic hydroperoxides, thereby protecting cells from oxidative damage(Brigelius-Flohé and Flohé 2020). Emerging evidence suggests that GPX2 may modulate ROS-mediated apoptosis pathways, thereby influencing the response to radiation therapy (You et al. 2023; Jiang et al. 2018). Zhou et al. found that ^125^I seed downregulates GPX2 expression in CCA cells by transcriptome sequencing (Zhou et al. 2022).

In light of these considerations, this study aims to investigate the therapeutic efficacy of ^125^I seed treatment in CCA cells and a rabbit VX2 CCA model and elucidate the underlying mechanisms, with a particular focus on the involvement of GPX2-mediated ROS-mediated apoptosis. Our findings provide valuable insights into the potential of ^125^I seed as a targeted therapeutic approach for CCA and highlight GPX2 regulation as a potential biomarker for treatment response.

## Materials and methods

### Cell culture and transfection

The normal bile duct cell line HIBEC and three CCA cell lines including QBC939, RBE and HuCCT1 were purchased from the Cell Bank of the Chinese Academy of Sciences (Shanghai, China). All cell lines were inoculated in 1640 medium (Sigma, USA) containing 10% fetal bovine serum (Gemini, USA), 1% penicillin and 1% streptomycin (Solarbio, China). Cells were cultured in an incubator at 37 °C with 5% CO2 volume fraction. For siRNA-mediated silencing of gene targets, GPX2 siRNA (siGPX2) and the lentiviruses with GPX2 overexpression were obtained from Gene Pharma (Shanghai, China). The lentiviral GPX2-expression constructs infected the CCA cells with polybrene (Sigma). The siRNA vectors transfected CCA cells using Lipofectamine RNAi Max (Invitrogen, Carlsbad, CA, USA) according to the manufacturer’s instructions. Transfection efficiency was determined by Western blotting.

### ^125^ I seed in vitro irradiation model

Model 6711 ^125^I seeds are manufactured by Ningbo Junan Pharmaceutical Technology Co., Ltd. The average energy of ^125^I seeds ranges from 27.2 to 34.6 keV, with a half-life of approximately 59.5 days. Experiments were conducted using 0.6 mCi (22.97 MBq) and 0.8 mCi (29.97 MBq) doses of ^125^I seeds. CCA cells (RBE and QBC939) were irradiated with different doses of ^125^I seeds for 96 h. The ex vivo irradiation model was established as described previously (Ma et al. 2011). This model consists of a lower irradiation plane and an upper cell culture plane, with a height of 6 mm between the irradiation plane and the cell culture plane. On the irradiation plane, eight seeds with the same activity are evenly distributed along a circumference with a diameter of 35 mm, with the ninth seed concentrated at the center. For the cells in the control group, sham seeds (without radioactivity) were used.

### Western blotting

First, proteins are extracted from a sample using RIPA buffer, and separation of protein samples by sodium dodecyl sulphate-polyacrylamide gel electrophoresis (SDS-PAGE). Then, the separated proteins are transferred from the SDS-PAGE to a nitrocellulose membrane. The nitrocellulose membrane is then blocked to prevent non-specific binding and incubated with primary antibodies specific to the protein of interest. After washing away unbound primary antibodies, the membrane is incubated with secondary antibodies. The membranes were finally exposed to chemiluminescence reagents and visualized using a chemidoc. The following primary antibodies were used for western blot analyses: anti-GPX2 (XY-KT-1700, xuanya), and GAPDH (ab8245, abcam).

### RNA isolation and quantitation

Total RNA was extracted using the TRIzol reagent (Cat. No. 15596026, Invitrogen) for reverse transcription-quantitative polymerase chain reaction (RT-qPCR). The extracted RNA was reverse transcribed into cDNA using the Ncode™ miRNA First-Strand cDNA Synthesis Kit (Thermo Fisher Scientific Inc., Waltham, MA, USA). The synthesized cDNA was then analyzed with the Fast SYBR Green PCR Kit (Applied Biosystems, Inc., Carlsbad, CA, USA). The GPX2 primer sequences were synthesized by Sangon Biotech (Shanghai, China). Primer sequences were 5’-TGCAACCAATTTGGACATCAG-3’ and 5’- AGACAGGATGCTCGTTCTGC-3’ for GPX2; 5’-CAGCCTCAAGATCATCAGCA-3’ and 5’-ATGATGTTCTGGAGAGCCCC-3’ for GAPDH.

### Histological analysis

The tumor tissue is fixed and embedded in paraffin wax. After deparaffinization and rehydration, sections are stained with hematoxylin and eosin (H&E). For immunohistochemistry (IHC), antigen retrieval and blocking of the sections are performed. Subsequently, the sections are incubated with appropriate primary and secondary antibodies. The signal is visualized using a DAB substrate (Pierce). The primary antibody used in IHC is anti-GPX2 (XY-KT-1700, xuanya), and Ki-67 (ab15580, Abcam).

### Clone formation assay

Harvested and processed RBE and QBC939 cells were reseeded at a concentration of 200 cells per well into 6-well plates. After culturing the cells for 1 week, they were stained with 0.05% crystal violet. Colony counts were calculated using an optical microscope from five or more different fields of view.

### Tunel assay

TUNEL assay (Roche Applied Bio Sciences, USA) (in vitro and *vivo*) was conducted to detect cellular apoptosis according to the manufacturer’s instructions. Briefly, the fixed samples were incubated with 100 µL of proteinase K for 30 min at 37 ℃. The slides were rinsed twice with PBS. The TUNEL reaction mixture was added to the samples and incubated at 37 ℃ for 60 min. Converter-POD solution was added to the samples and incubated at 37 ℃ for 30 min. The results were analyzed under a fluorescent microscope (Olympus FluoView™ FV1000, Tokyo, Japan). Image and data acquisition and analysis were performed using analysis software (Image Pro Plus, Media Cybernetics, USA).

### Detection of ROS

ROS levels were determined using the fluorescent dye dihydroethidium (DHE, Beyotime Biotech) and were performed according to a previously reported protocol (Chen et al. 2021). Briefly, CCA cells were incubated with DHE (10 µmol/L) at 37 °C in the dark for 30 min. DHE was excited with a 488 nm argon laser. The method for detecting ROS levels in VX2 rabbit tumor tissue was similar to cells by using frozen slides. Three random fields were selected for analysis under a fluorescence microscope (Leica).

### Animal model

Ethical approval was obtained from our Hospital Research Ethics Committee consistent with the ethical guidelines of the 1975 Declaration of Helsinki (Ethics No. B2022-364R). Forty healthy New Zealand White rabbits, regardless of sex, weighing 2–2.5 kg and aged 4–5 months, were provided and housed by the Animal Center of Guangzhou Medical University. Among them, three were used for tumor-bearing rabbits. The rabbit VX2 CCA model was established, with VX2 tumor cells provided by Zhongshan Medical University. Firstly, tumors were dissected from the tumor-bearing rabbits, and the tumor tissue was minced into pieces of approximately 1 mm^3^ in size for later use. Subsequently, the rabbits were anesthetized and placed in a supine position on the operating table. A midline incision of approximately 4–5 cm along the linea alba below the xiphoid process was made, and the stomach and duodenum were gently exposed, followed by the identification of the bile duct along the gallbladder. Then, 1 ml of tumor tissue mass suspension was injected into the bile duct lumen, and the needle was slowly withdrawn upon observing a small bulge. Finally, the abdomen was closed layer by layer.

### ^125^ I implantation

Two weeks later, after confirming the diagnosis of CCA via PET-CT, rabbits with CCA were randomly assigned to three groups: the sham group with sham seed implantation, the 0.6 mCi group receiving implants of 0.6 mCi ^125^I seed, and the 0.8 mCi group receiving implants of 0.8 mCi ^125^I seed. Under sterile conditions, the abdomen was opened to expose the implanted VX2 tumors. According to the predetermined needle insertion pathway, ^125^I seeds and sham seeds were respectively implanted into the visible masses within the bile duct.

### PET/CT imaging

PET/CT (GE DiscoveryLS) and 18 F-fluorodeoxyglucose (FDG) were provided by Shanghai Kexin Electronic Technology Co. Ltd. and Huashan Hospital of Fudan University, respectively, and the radiochemical purity of 18 F-FDG was > 95%. The experimental animals were fasted for 6 h and anesthetized with 3% pentobarbital. 18 F-FDG was injected intravenously at 0.5 mCi/kg of body mass from the ear margin, and the computer synchronously started the dynamic acquisition. The CT acquisition conditions were as follows: voltage, 140 kV; current, 160 mA; pitch, 5.0 mm; spiral time, 0.8 s/circles; layer thickness 4.25 mm. The PET images were acquired in the 2D mode, and the time for each bed was 5 min, and the PET images were attenuated and corrected by the CT data after the completion of the acquisition. PET images were reconstructed using the ordered subset maximum expected value iterative method. CT reconstruction was performed using the standard reconstruction method with a matrix of 512 × 512 and a layer thickness of 5 mm. Image fusion was performed using the Xeleris software on the workstation. ^125^I (0.6 mCi and 0.8 mCi) seeds were implanted into the tumor site of VX2 rabbits. 30 days later, PET-CT scans were performed to obtain a tumor index.

### Statistical analysis

The data are presented as mean ± standard error of the mean (SEM). Statistical analyses were performed using Prism 6 (GraphPad Software, San Diego, CA, USA) with Student’s t-test or one-way analysis of variance (ANOVA). Significance levels were denoted as follows: *P < 0.05 and #*P* < 0.05. Each result was replicated at least three times.

## Results

### The ^125^I seeds inhibit proliferation and promote ROS generation

**Fig. 1:**
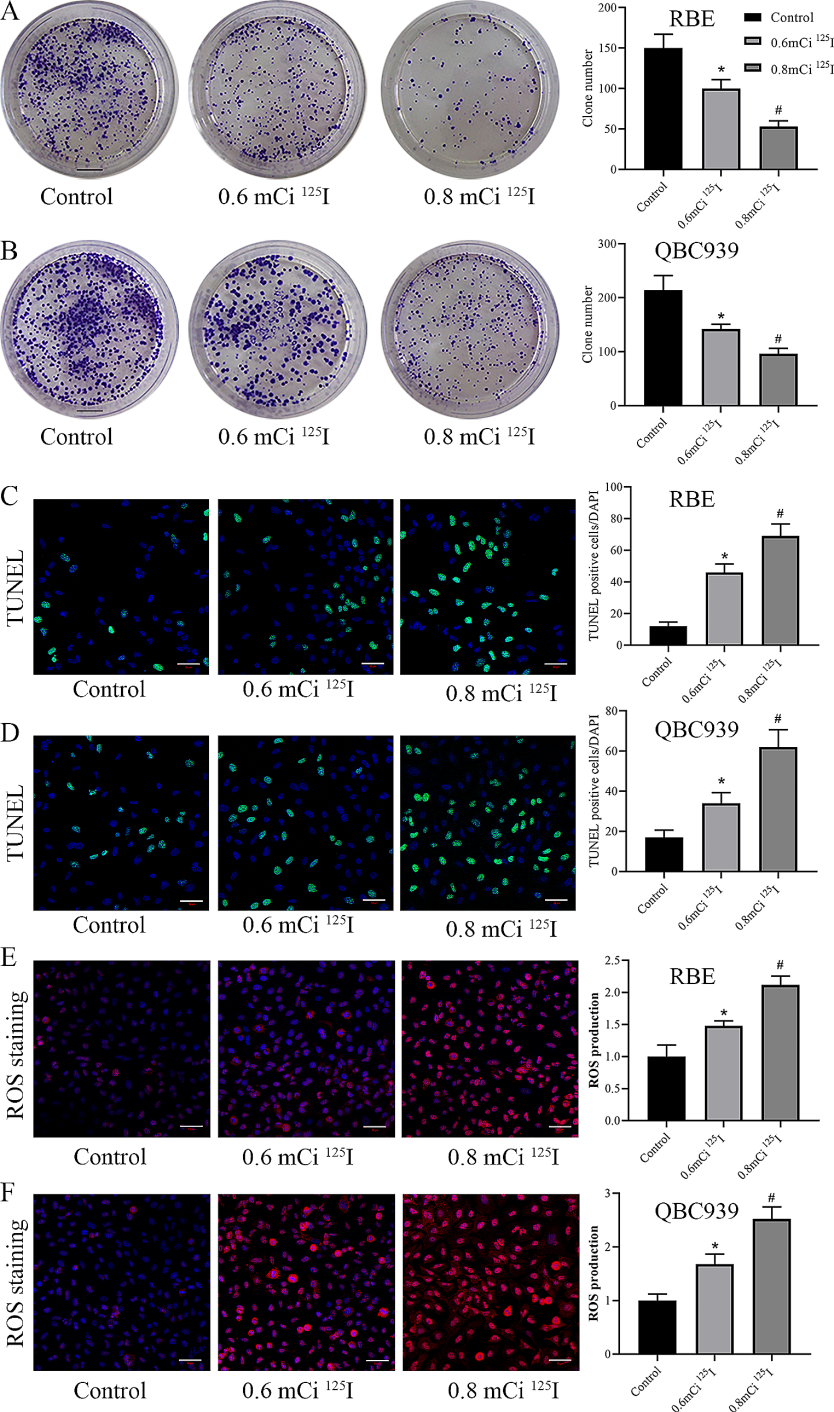
The ^125^I seeds inhibit proliferation and promote ROS generation. **A** The cell growth in RBE cells was measured using colony formation assay. **B** The cell growth in QBC939 cells was measured using colony formation assay. Scale bar: 1 cm. **C** Tunel staining assay for apoptosis in RBE cells. **D** Tunel staining assay for apoptosis in QBC939 cells. Scale bar: 50 μm. **E** Representative images of ROS detected by DHE and a summary of the relative fluorescence intensity of ROS in each group of RBE cells. **F** Representative images of ROS detected by DHE and a summary of the relative fluorescence intensity of ROS in each group of QBC939 cells. Scale bar: 50 μm. *P < 0 0.05 vs. control group, #*P* < 0.05 vs. 0.6 mCi ^125^I group

To investigate the impact of ^125^I seeds on the process of CCA, we detected CCA cell proliferation using clone formation assay. The results indicate that compared to control cells, the clone formation rate of RBE cells decreases when the dose of ^125^I seeds is 0.6 mCi, and further decreases when it reaches 0.8 mCi (Fig. 1A). Similarly, compared to control cells, the clone formation rate of QBC939 cells decreases with increasing dose of ^125^I seeds in both the 0.6 mCi and 0.8 mCi groups (Fig. 1B). Tunel assay showed that ^125^I significantly elevated the apoptosis level of RBE cells and QBC939 cells, and the pro-apoptotic effect was strongest at 0.8 mCi compared to 0.6 mCi (Fig. 1C-D). Subsequently, we examined the levels of intracellular ROS through DHE staining. The results reveal that compared to controls, the levels of ROS increase in RBE cells when the dose of ^125^I seeds is 0.6 mCi, and this increase becomes more significant at 0.8 mCi (Fig. 1E). Likewise, similar results were observed in QBC939 cells (Fig. 1F). Overall, ^125^I seeds inhibited CCA cell proliferation and promoted intracellular ROS generation in a dose-dependent manner in vitro.

### Decreased GPX2 expression promotes ROS-mediated apoptosis in vitro

**Fig. 2:**
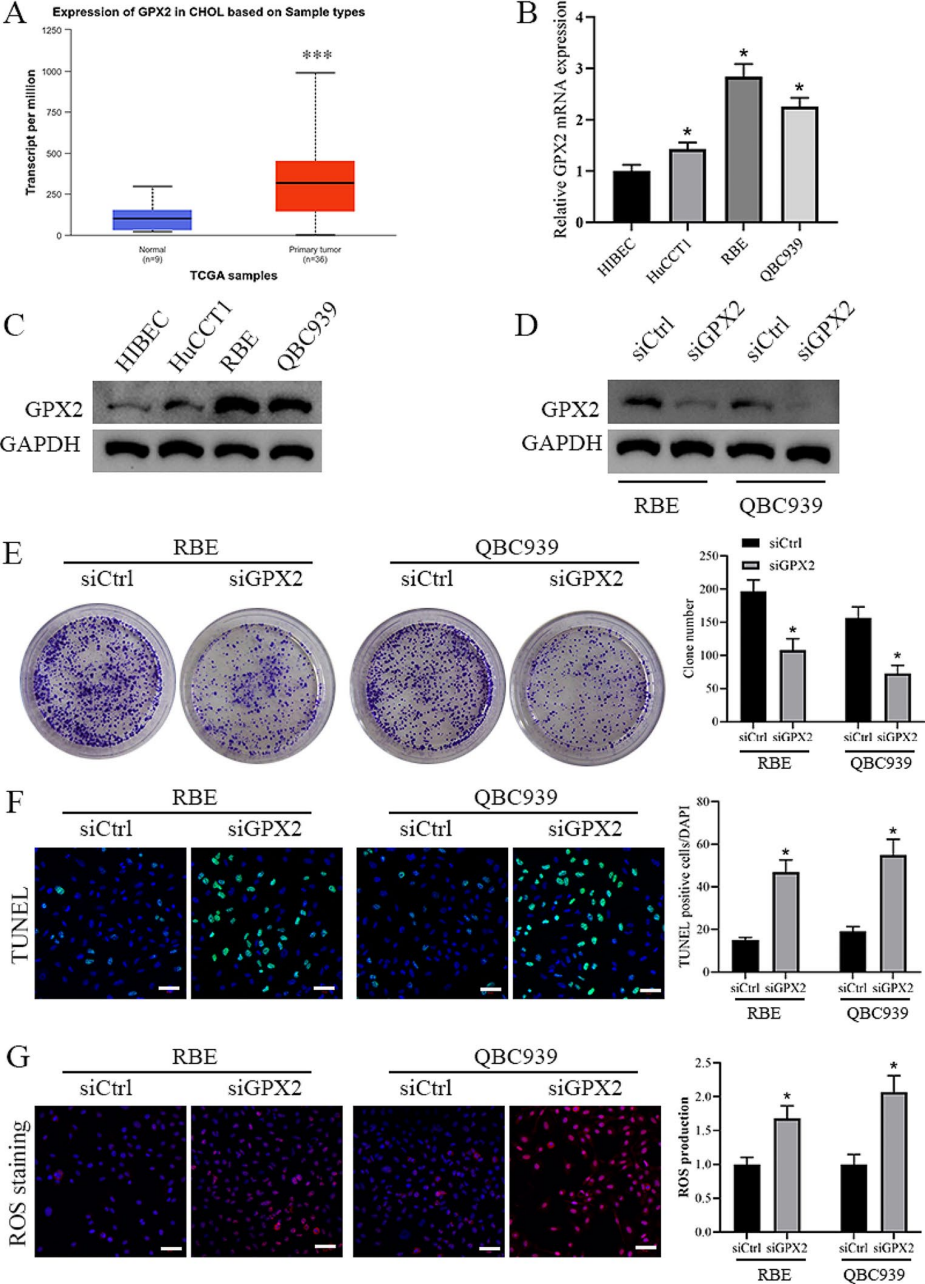
Decreased GPX2 expression promotes ROS-mediated apoptosis in vitro. **A** Transcriptional levels of GPX2 in CCA compared to normal tissues. Data were obtained from the UALCAN database (http://ualcan.path.uab.edu/index.html). **B** Quantitative RT-PCR-detected levels of GPX2 mRNA expression in normal bile duct cell line, HIBEC, and five CCA cell lines. **C** Western blot analysis of GPX2 protein expression levels. **D** Western blot results showed that GPX2 was markedly inhibited by GPX2-specific siRNA. **E** Effect of ^125^I seeds on the colony formation of CCA cells. Scale bar: 1 cm. **F** The cell apoptosis was measured by TUNEL staining. Scale bar: 50 μm. **G** Detection of intracellular ROS levels by DHE staining. Scale bar: 50 μm. *P < 0 0.05 vs. siCtrl group

We analyzed the TCGA database and identified significant upregulation of GPX2 expression in CCA (Fig. 2A). Subsequently, we validated GPX2 expression in CCA cell lines. Our findings revealed that both mRNA and protein levels of GPX2 were notably elevated in the three CCA cell lines compared to HIBEC (Fig. 2B-C). To further elucidate the pivotal role of GPX2 in CCA, we employed GPX2-specific siRNA to significantly inhibit GPX2 expression in RBE and QBC939 cells (Fig. 2D). Remarkably, the downregulation of GPX2 resulted in a significant inhibition of proliferation and an enhancement of apoptosis in both RBE and QBC939 cells (Fig. 2E-F). Consistent with these findings, intracellular ROS levels were markedly elevated upon GPX2 knockdown in both cell lines (Fig. 2G). These results suggest that the downregulation of GPX2 in CCA promotes apoptosis via ROS modulation.

### GPX2 played an important role in apoptosis induced by ^125^I seeds in vitro

**Fig. 3:**
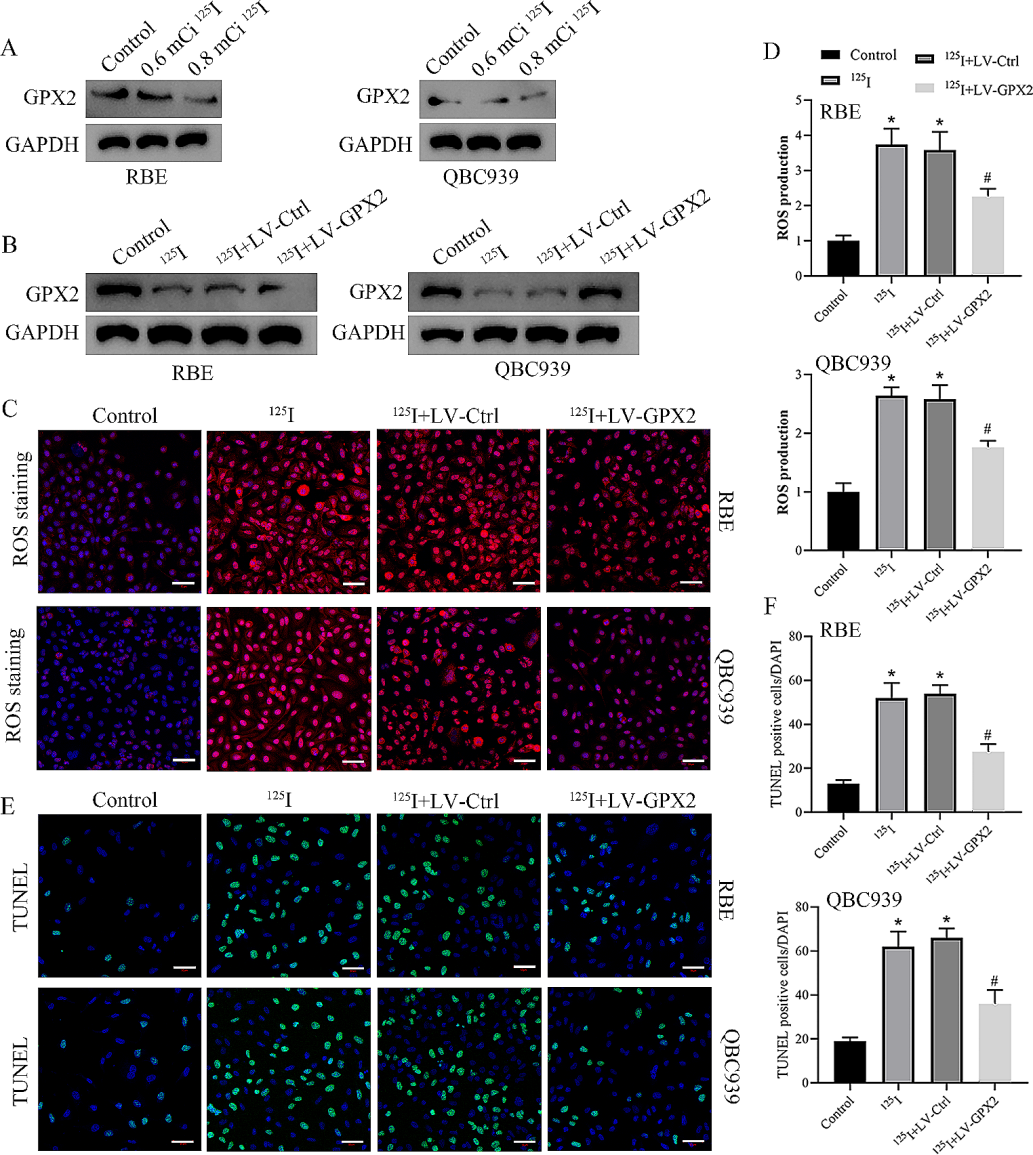
GPX2 played an important role in apoptosis induced by^125^I seeds in vitro. **A** Western blotting to detect the effect of ^125^I seeds on GPX2 expression. **B** GPX2 expression of cells in each group was detected using Western blotting. **C, D** Detection of intracellular ROS levels by DHE staining. Scale bar: 50 μm. **E, F** The cell apoptosis was detected by TUNEL assay. Scale bar: 50 μm. *P < 0 0.05 vs. control group, #*P* < 0.05 vs.^125^I+LV-Ctrl group

To investigate the role of GPX2 in ^125^I seed-induced anticancer effects on CCA. We examined the changes in GPX2 expression in CCA cells after ^125^I seed treatment. Western blot analysis revealed that the expression levels of GPX2 in the 0.6 mCi ^125^I group were significantly lower than those in the control group. Additionally, compared to the 0.6 mCi ^125^I group, GPX2 expression was significantly decreased in the 0.8 mCi ^125^I group (Fig. 3A). CCA cells were treated with 0.8 mCi ^125^I seeds in the following experiments. Overexpression of GPX2 in ^125^I group upregulates GPX2 levels (Fig. 3B). Overexpression of GPX2 markedly reversed the promoting effect of ^125^I seeds on intracellular ROS generation in CCA cells (Fig. 3C-D). TUNEL analysis also demonstrated that overexpression of GPX2 could reverse the ability of ^125^I seeds to promote apoptosis in CCA cells (Fig. 3E-F). Our findings indicate that ^125^I seeds promote ROS-mediated apoptosis by regulating GPX2.

### ^125^I seed implantation inhibited tumor growth in rabbit VX2 tumor transplantation model

**Fig. 4:**
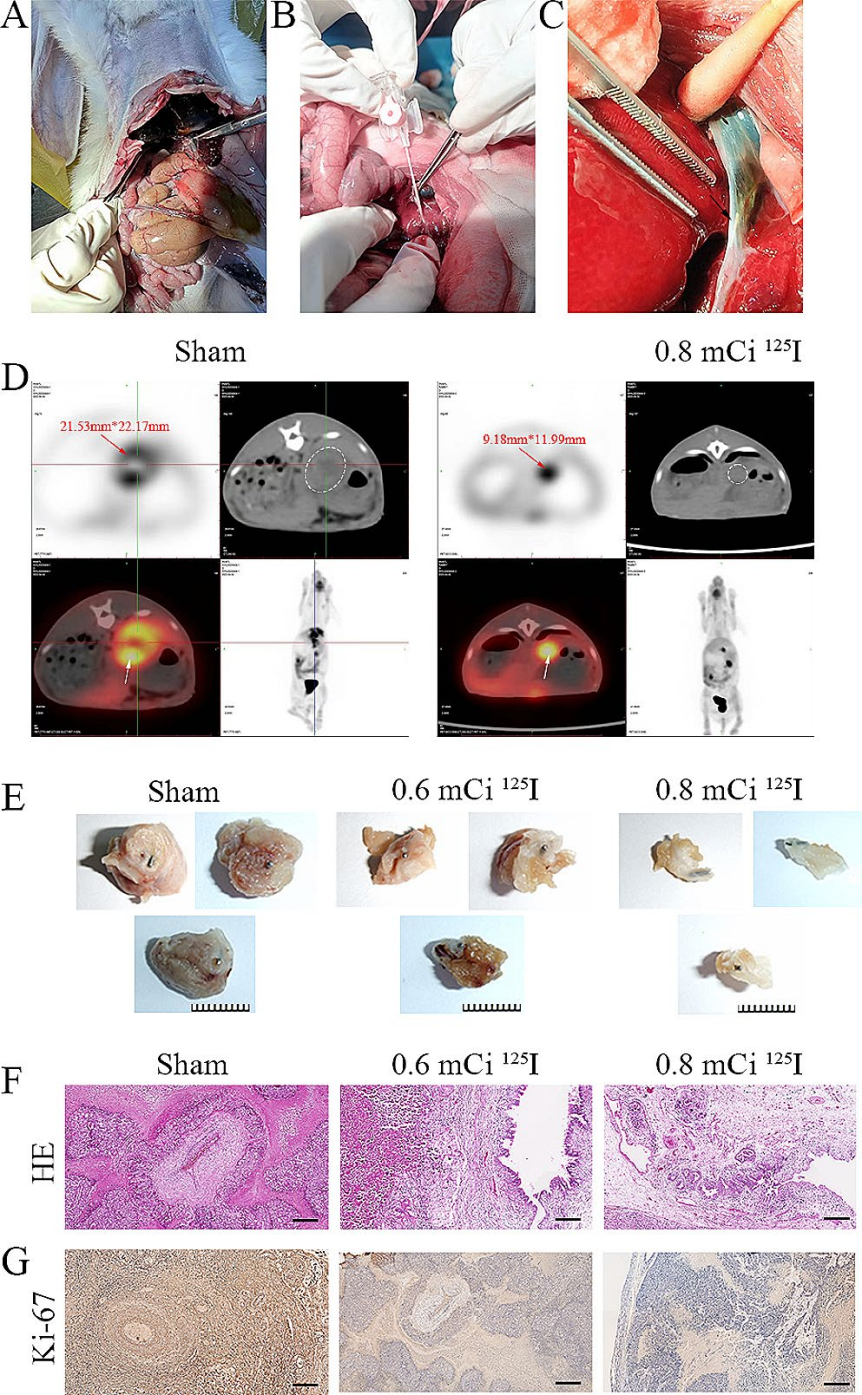
^125^I seed implantation inhibited tumor growth in rabbit VX2 tumor transplantation model. **A** A midline incision of approximately 4–5 cm along the linea alba below the xiphoid process, gently exposing the stomach and duodenum, then tracing the common bile duct downward along the gallbladder. **B** Tumour tissue mass suspension is injected into the lumen of the bile ducts. **C** After tumor formation in vivo, the abdomen was reopened and ^125^I seeds were implanted in the bile ducts. Arrows indicate ^125^I seed. **D** Representative PET-CT imaging of tumor-bearing rabbit. Arrows or circles indicate a tumor. **E** Photograph of dissected tumors. **F** Representative images of H&E staining. Scale bar: 1 cm. G The protein expression of Ki-67 in dissected tumor samples was evaluated by IHC. Scale bar: 50 μm. *N* = 3

To establish the tumor-bearing rabbit model, VX2 cells were suspended and injected into the thigh muscles of rabbits. Leg swelling was monitored daily until successful tumor engraftment was confirmed. Following the establishment of the VX2 CCA model, intra-biliary duct implantation of ^125^I seed was performed (Fig. 4A-C). PET-CT scans of the upper abdomen were conducted 30 days after ^125^I seed implantation to observe tumor size. The results demonstrated a significant reduction in rabbit CCA burden following ^125^I seed implantation (Fig. 4D). Consistently, a marked decrease in tumor volume was observed in rabbits implanted with ^125^I seed in a dose-dependent manner (Fig. 4E). Pathological analysis indicated a significantly lower percentage of tumor area in these rabbits (Fig. 4F). Immunohistochemical (IHC) staining of Ki-67 revealed suppression of CCA cell proliferation by ^125^I seed, while 0.8 mCi had the strongest inhibitory effect compared with 0.6 mCi (Fig. 4G). In summary, our data indicate potent tumor-suppressive effects of ^125^I seed in vivo.

### Mechanism of^125^I seed implantation therapy in vivo

**Fig. 5:**
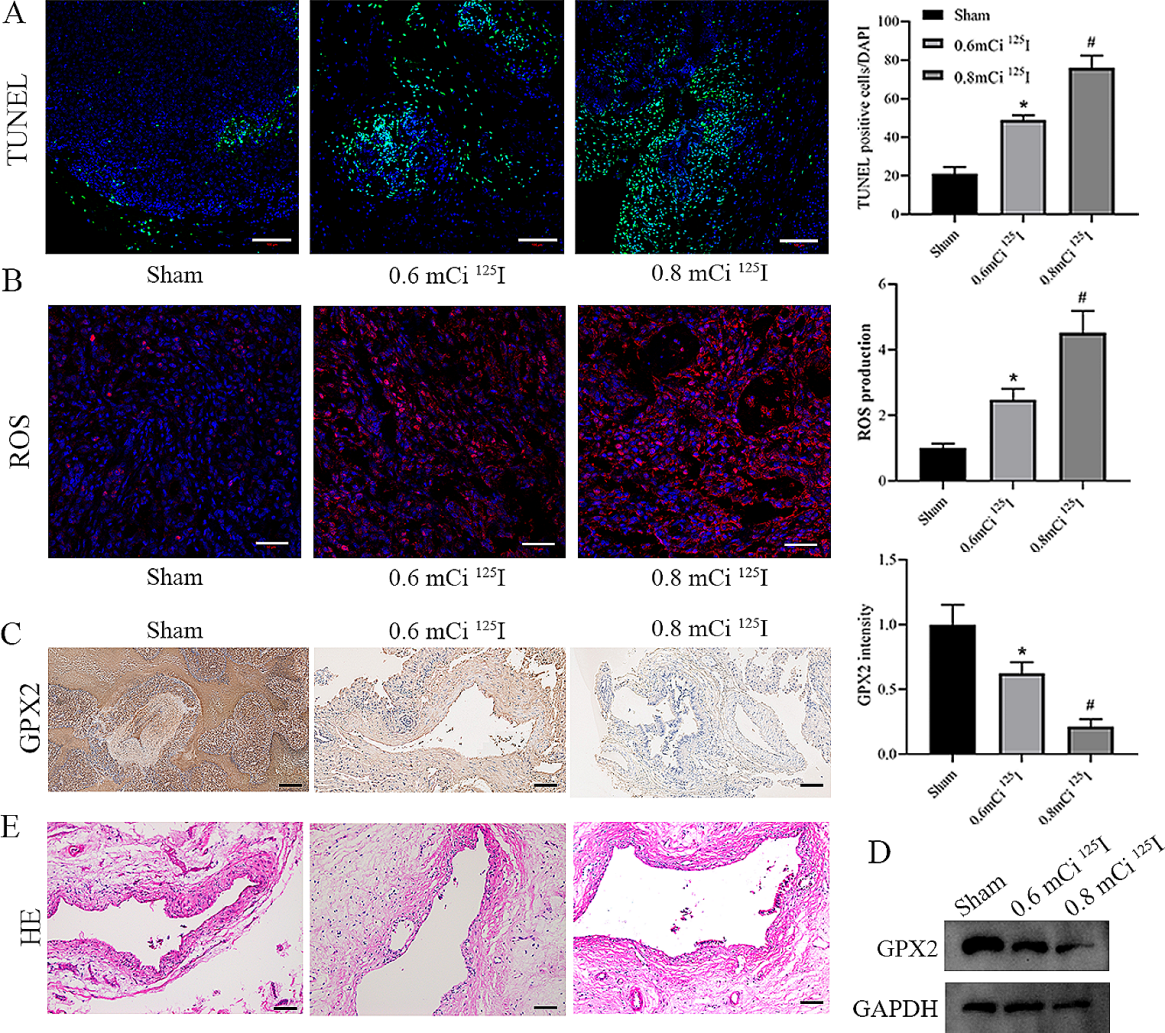
Mechanism of ^125^I seed implantation therapy in vivo. **A** Represent images of the resected tumors stained with TUNEL in each group.Scale bar: 100 μm. **B** DHE staining to detect ROS levels in frozen slides of tumor tissue. Scale bar: 50 μm. **C** Represent images of the resected tumors stained with GPX2 in each group. Scale bar: 50 μm. **D** Western blotting to detect GPX2 expression in the VX2 CCA model. **E** Observation of bile duct wall samples under a light microscope. Scale bar: 50 μm. *N* = 3. *P < 0 0.05 vs. sham group, #*P* < 0.05 vs. 0.6 mCi ^125^I group

Finally, we further validated the regulatory role of ^125^I seeds on the GPX2-mediated ROS-related apoptosis pathway in vivo. TUNEL experiments revealed a significant increase in apoptosis levels within tumor tissues after ^125^I seed implantation. Compared to the 0.6 mCi ^125^I group, the 0.8 mCi ^125^I group exhibited a more pronounced increase in apoptosis levels (Fig. 5A). Consistently, ROS levels increased as the ^125^I seed dose increased (Fig. 5B). IHC analysis detected the expression of GPX2 in tumor tissues. The results showed a weakening of GPX2-positive staining in tumor tissues after ^125^I seed implantation compared to the control group (Fig. 5C). Western blotting results further confirmed that ^125^I seed downregulates GPX2 expression in a dose-dependent manner (Fig. 5D). Interestingly, H&E staining revealed that ^125^I seed implantation did not result in significant bile duct wall damage (Fig. 5E). In summary, ^125^I seed promotes ROS-mediated apoptosis of CCA cells by inhibiting the expression of GPX2.

## Discussion

CCA is a relatively rare type of malignant tumor originating from the epithelium of the bile ducts, which can occur in any segment of the biliary system (Blechacz et al. 2011). Due to the lack of specific symptoms in the early stage of CCA, the lack of standard and effective early screening tools, the narrow anatomical space of the bile ducts, and the proximity of important hepatic vascular structures, most of the CCA have already lost the chance of radical surgery when diagnosed (Saengboonmee et al. 2018; Vogel et al. 2014). For patients who cannot undergo surgical resection or lose the chance of surgery in the middle and late stages, ^125^I seed implantation therapy undoubtedly opens up a new therapeutic pathway (Wang et al. 2013; Sun et al. 2018). In this study, we investigated the therapeutic potential of ^125^I seed implantation therapy as an alternative treatment modality for CCA, focusing on its ability to induce ROS-mediated apoptosis and the involvement of GPX2 in this process.

Our findings demonstrate that ^125^I seeds effectively inhibit the proliferation of CCA cells in vitro. Support for this was also given by the result of experimental analysis using the rabbit VX2 CCA model. The VX2 rabbit cancer model has been applied to multiple organs, including the liver (Pascale et al. 2022), kidney (Li et al. 2019a, b), and brain (Wang et al. 2015). A previous study described the implantation of VX2 tumors into rabbit bile ducts (Suzuki et al. 1994). In our animal study, VX2 tumors were successfully grown in rabbit bile ducts. All rabbits tolerated both VX2 implantation and ^125^I seed treatment. Importantly, molecular analyses revealed a significant increase in ROS levels in vivo and in vitro following treatment with ^125^I seed, indicating the activation of ROS-mediated apoptotic pathways. ROS, known for their dual roles as signaling molecules and cytotoxic agents, play a crucial role in modulating cellular responses to radiation therapy (Sahoo et al. 2022). This aligns with previous research demonstrating that combined treatment of ^125^I seed and salinomycin achieved enhanced growth inhibition and apoptosis in human glioma in vitro and in vivo through triggering ROS-mediated apoptosis (Chao et al. 2018; Chen and Wang 2017). ^125^I seed radiation‑induced apoptosis, paraptosis and autophagy were considerably mediated by ROS in human esophageal squamous cell carcinoma (Wang et al. 2020). The induction of ROS-mediated apoptosis represents a key mechanism underlying the therapeutic effects of ^125^I seed implantation in CCA.

Furthermore, our study sheds light on the role of GPX2 in modulating ROS-mediated apoptosis in CCA. GPX2, a key antioxidant enzyme, is involved in the detoxification of ROS and protection against oxidative stress-induced damage. The oncogenic effects of GPX2 have been demonstrated in a variety of cancers and are associated with tumor progression (Wang et al. 2019; Naiki et al. 2014). Interestingly, we observed that GPX2 expression was up-regulated in CCA and downregulation of GPX2 following treatment with ^125^I seed, suggesting its potential role in sensitizing CCA cells to ROS-mediated apoptosis. This finding is in line with previous studies implicating GPX2 in the regulation of cellular responses to oxidative stress and radiation therapy (Tan et al. 2023). Inhibiting GPX2 could increase ROS levels and enhance the anti-tumor effect of lenvatinib (Tan et al. 2023). GPX2 is a direct target of p63 and inhibits oxidative stress-induced apoptosis in breast cancer cells in a p53-dependent manner(Yan and Chen 2006). YAP promoted ROS accumulation through the downregulation of GPX2, thus triggering cell growth inhibition in lung squamous cell carcinoma (Huang et al. 2017).

There are still some shortcomings in the study. Firstly, although H&E staining showed that ^125^I seeds did not cause significant damage to the bile duct wall, the overall safety assessment was not comprehensive enough. The potential side effects and long-term effects of ^125^I seeds on other normal tissues and organs were not assessed in detail. In addition, although the study revealed the role of GPX2 in ROS-mediated apoptosis, the specific regulatory pathways of GPX2 in apoptosis and other molecular mechanisms that may be involved were not elaborated. Finally animal experiments did not involve GPX2 overexpression. We will improve these deficiencies in subsequent experiments.

Overall, our study provides mechanistic insights into the therapeutic efficacy of ^125^I seed implantation therapy in CCA. The induction of ROS-mediated apoptosis and the involvement of GPX2 highlight promising avenues for further research and therapeutic intervention. Targeting ROS-mediated apoptotic pathways and modulating GPX2 expression may represent novel strategies to enhance the effectiveness of ^125^I seed implantation therapy and improve outcomes for patients with CCA. Further preclinical and clinical studies are warranted to validate these findings and explore their translational potential in the clinical management of CCA.

## Data Availability

No datasets were generated or analysed during the current study.
