# Correction: Mechanistic insights into ^125^I seed implantation therapy for Cholangiocarcinoma: focus on ROS-Mediated apoptosis and the role of GPX2

**DOI:** 10.1007/s00432-025-06111-2

**Published:** 2025-02-13

**Authors:** Jun Luo, Zheng Yao, Weiren Liang, Danjun Song, Hui Zeng, Yi Jiang, Zhehan Bao, Jiaping Zheng, Yinan Ding

**Affiliations:** Department of Interventional Radiology, Zhejiang Cancer Hospital, Hangzhou Institute of Medicine (HIM), Chinese Academy of Sciences, Zhejiang Key Laboratory of Imaging and Interventional Medicine, Hangzhou, Zhejiang 310022 China

**Correction: Journal of Cancer Research and Clinical Oncology (2024) 150:324**

10.1007/s00432-024-05840-0

In this article the affiliation details for the entire author group were incorrectly given as ‘Department of Interventional Radiology, Hangzhou Institute of Medicine (HIM), Zhejiang Cancer Hospital, Chinese Academy of Sciences, Hangzhou, Zhejiang 310022, China’ but should have been ‘Department of Interventional Radiology, Zhejiang Cancer Hospital, Hangzhou Institute of Medicine (HIM), Chinese Academy of Sciences, Zhejiang Key Laboratory of Imaging and Interventional Medicine, Hangzhou, Zhejiang 310022, China’.

The original article has been corrected.

